# Gender Differences in Anger Among Hospital Medical Staff Exposed to Patients with COVID-19

**DOI:** 10.1089/heq.2020.0119

**Published:** 2021-04-19

**Authors:** Ulrich Wesemann, Nino Hadjamu, Reza Wakili, Gerd Willmund, Julia Vogel, Tienush Rassaf, Johannes Siebermair

**Affiliations:** ^1^ Department of Psychiatry, Psychotherapy and Psychotraumatology, Bundeswehr Hospital, Berlin, Germany.; ^2^ Department of Cardiology and Vascular Medicine, West German Heart and Vascular Center Essen, University Duisburg-Essen, Essen, Germany.

**Keywords:** COVID-19, anger, mental health, PTSD, medical staff, hospital, gender

## Abstract

**Purpose:** Occupational exposure to patients with COVID-19 is a stress factor. The aim of this study was to assess gender differences in anger among medical hospital staff.

**Methods:** *N*=78 hospital employees with direct or indirect contact to patients with COVID-19 completed State-Trait Inventory-2.

**Results:** Female personnel showed higher scores in the main “trait anger” scale and its subscale “anger temperament,” whereas “anger control-out” was significant lower. Direct patient contact had no influence.

**Conclusion:** More specific training for female hospital staff could achieve health-related equity. Focusing on anger as a leading indicator could lead to better prevention and self-monitoring. Registered at Clinicaltrials.gov (NCT04368312).

## Introduction

Exposure to critical events can be seen as a stress factor, the impact of which may depend on the proximity to the stressor,^1–3^ personal risk perception,^4^ gender,^5,6^ or occupation.^7^ Vulnerability, resilience, and the current mental state are also considered relevant,^8–10^ but are rarely recorded before disasters. A large study found incident rates for depression (54%), anxiety (45%), insomnia (34%), and distress (72%) among health care workers who treated patients with COVID-19.^2^ These results are supported by a recently published review of meta-analyses.^11^ In high-risk hospital patients with COVID-19 and severe pulmonary, cardiovascular, or oncological diseases, the prevalence rate for post-traumatic stress disorder (PTSD) was 38%.^12^

In North Rhine-Westphalia in Germany, the cumulative incidence of COVID-19 up to June 4 was 215 per 100,000 population with a mortality rate of 4.2%. Therefore, COVID-19 was a serious threat at that time. This can lead to an impairment of the mental health of the hospital staff who treat the affected patients. The city of Essen reported 874 cases with a COVID-19 infection until June 4. At the University Hospital Essen >200 patients with COVID-19 were hospitalized between March and June 2020. During this period, medical staff were routinely exposed to patients with COVID-19.

The effects on mental health of hospital employees in response to a pandemic differ individually and very likely depending on the proximity to patients with COVID-19.^2,13^ These treatments cannot be replaced by telemedicine or online treatment, as is the case with psychotherapy^14^ or the remote monitoring of patients with an implantable cardioverter-defibrillator.^15^

In a different context, female gender has been found to be a predictor of the onset of PTSD among military health care personnel after deployment.^16^ Anger is a common response to work-related life-threatening situations.^17^ Female gender has also been reported to be an important indicator of the increasing anger of military medical personnel, which can be seen as an emotional indicator of the later occurrence of PTSD, anxiety disorders, or depression.^18^ The aim of this study was to assess gender differences in expressed anger of medical hospital staff working with patients with COVID-19. Owing to the more frequent double burden on work and family life among German female workers, higher scores are expected for female workers.

## Methods

A total of *N*=78 hospital employees from the Department of Cardiology and Vascular Medicine as well as from the Center of Emergency Medicine participated in the study, including *n*=52 female (67%) and *n*=26 (33%) male individuals. Mean age was 32.7±SD 9.38 years and a mean work experience 8.1±SD 9.25 years. The proximity of medical staff to patients with COVID-19 was assessed on a ranking scale, giving participants with direct patient contact (*n*=40; 51%), participants with patient contact with suspected COVID-19 infection (*n*=8; 10%), and participants without direct contact to COVID-19 diagnosed patients (*n*=30; 39%).

The sample included medical specialists in cardiology and anesthesia (*n*=11; 14%), residents (*n*=30; 39%), and nurses (*n*=37; 47%). Participation was possible for all employees in these sections, it was voluntary and written informed consent was obtained from all individuals. The data acquisition took place between April 2 and May 15. This was 4–12 weeks after admission of the first patients with COVID-19 to the hospital. The study was approved by the local Ethics Committee of the University of Essen Medical School (IRB No. 20-9263-BO).

Participants completed questionnaires for sociodemographic data and the State-Trait Anger Expression Inventory-2 (STAXI-2). The STAXI-2 consists of 51 questions to record various aspects of anger on a 4-point Likert scale. Situation-related anger (state anger) is measured as well as various dispositional dimensions of anger: characteristic anger with the components anger temperament and anger reaction, forms of anger expression (inner or outer anger expression), and anger control.^19^

To exclude sample bias, relevant psychometric and sociodemographic parameters from female and male participants were compared with one-way ANOVAs for metric and Pearson's *χ*^2^-tests for categorical parameters. One-way ANOVAs were performed to test gender differences in the STAXI-2 scales. Afterward, ANCOVAS controlling for occupation, proximity to the patients with COVID-19, age, and work experience were performed. Finally, Pearson correlations were carried out because STAXI-2 scales have an interval level and the gender, in this case as a dichotomous variable, is metrically scaled. Statistical analyzes were carried out with SPSS (version 21; IBM, Inc., Armond, NY). The statistical significance level was set to *p*<0.05; due to the pilot nature of the study, no alpha correction was carried out.

## Results

There was no difference in work experience, age, or proximity to patients with COVID-19 between female and male gender, except for the professional group with more female nurses (*F*_1, 76_=9.41; *p*=0.003). One-way ANOVA revealed significant differences in the main trait anger scale (frequency of angry feelings over time; *F*_1, 72_=5.10; *p*=0.027) and its subscale anger as a temperament (*F*_1, 71_=4.72; *p*=0.033), with higher scores in females. Anger control-out (control of angry feelings by preventing the expression of anger toward other people or objects in the area) was significant lower in females (*F*_1, 69_=8.66; *p*=0.004). Homoscedasticity was satisfied for all significant results. Results of all scales and subscales are provided in Table 1.

**Table 1.** Mean Values, Standard Deviations, and One-Way Analysis of Variance in Anger Scales from STAXI-2 for Comparison of the Genders

Measure	Female		Male		F (1, 68–72)	η^2^
	M	*SD*	M	*SD*		
State anger	18.41	6.66	17.50	4.68	0.36	0.01
Feeling of anger (state)	6.62	3.47	6.25	1.96	0.23	0.00
Verbal anger impulse (state)	6.47	2.80	6.04	2.40	0.41	0.01
Physical anger impulse (state)	5.22	0.52	5.21	0.72	0.01	0.00
Trait anger	21.39	6.62	18.04	4.63	5.10^*^	0.07
Anger temperament (trait)	9.58	3.72	7.76	2.67	4.72^*^	0.06
Anger reaction (trait)	10.08	2.81	8.96	3.12	2.45	0.03
Anger expression-out	11.98	2.77	11.12	3.22	1.39	0.02
Anger expression-in	15.39	5.09	14.04	5.53	1.08	0.02
Anger control-out	14.24	2.89	16.24	2.42	8.66^**^	0.11
Anger control-in	13.73	2.89	12.68	3.93	1.68	0.02

^*^ *p*<0.05; ^**^p<0.01.

STAXI-2, State-Trait Anger Expression Inventory-2.

**FIG. 1.**
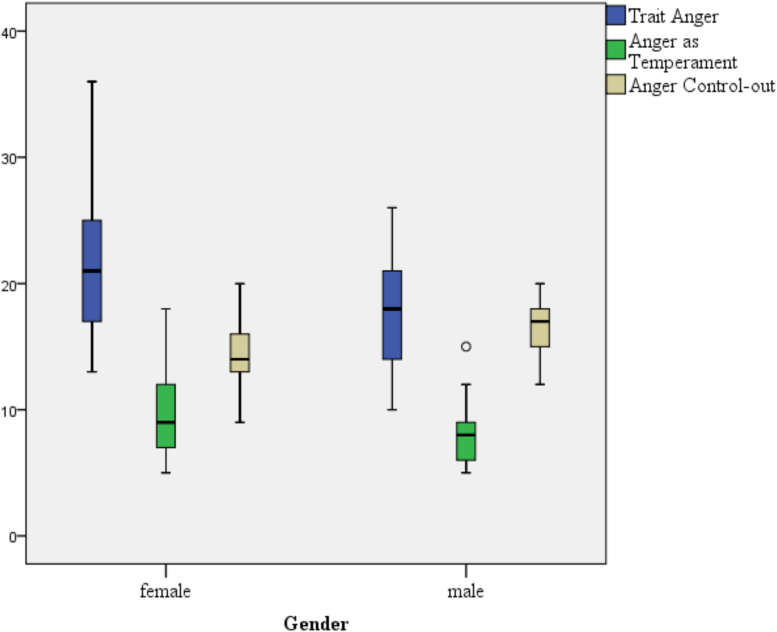
Boxplot with significantly higher values in “Trait Anger” and “Anger as Temperament” as well as lower values in “Anger Control-out” for female medical staff compared with their male colleagues during the time of COVID-19.

ANCOVAs confirmed the influence of gender on the earlier identified anger scales when controlling for occupation, proximity to the patients with COVID-19, as well as age and work experience. A correlation between gender and the main trait anger scale (*r*=−0.26; *p*=0.027), the subscale anger as a temperament (*r*=−0.25; *p*<0.033), and anger control-out (*r*=0.33; *p*=0.004) confirmed the results. An overview of all correlations is provided in Table 2. Figure 1 summarizes the results graphically by quartile using a boxplot.

**Table 2.** Bravais–Pearson Correlation Matrix Between Gender and STAXI-2 Scales

Variable	Feeling of anger^a^	Verbal anger impulse^a^	Physical anger impulse^a^	Anger temperament^b^	Anger reaction	Anger expression-out	Anger expression-in	Anger control-out	Anger control-in	State anger	Trait anger
Gender^c^	−0.06	−0.08	−0.01	−0.25^*^	−0.18	−0.14	−0.12	0.33^**^	−0.15	−0.07	−0.26^*^

*N*=63.

^a^ State variable.

^b^ Trait variable.

^c^ Available options=male, female, diverse.

^*^ Indicates *p*<0.05. ^**^Indicates *p*<0.01.

## Discussion

The results support the hypothesis that female hospital workers develop a higher level of anger when exposed directly or indirectly to patients with COVID-19. This is in line with previous research that focuses on other threatening stimuli.^16,18,20^ Regarding the theory of tokenism,^21^ more frequent harassment of minorities in male-dominated professions has been given as an explanation for these results. In this study, the more common double burden of work and family for female employees is seen as an explanation. During this time, the workload of hospital staff was higher, although kindergartens and schools were closed.

Nevertheless, this study has several limitations. The sample size is small, there is no baseline information, more female staff are nurses, and due to the pilot character of the study, no control for multiple testing was included. Therefore, the results should be considered preliminary and pending validation through a confirmation study. Confirming the results could lead to more specific training for female hospital staff to achieve health-related equity, as anger is also related to serum concentrations of TNF-α and its soluble receptors.^22^ This could also help avoid the “finger pointing” that became common after the COVID-19 outbreak,^23^ by following basic ethical issues.^24^ This could reduce negative emotional contagion, which has been shown to be an important predictor of negative mental health effects after trauma.^25^

Aside from prevention, focusing on anger as a leading indicator of later occurrence of psychological symptoms could lead to better detection of psychological impairment and self-monitoring.

## Declarations

### Availability of data and material

The data that support the findings of this study are available from the corresponding author upon reasonable request.

## Abbreviations Used

PTSD: post-traumatic stress disorder
STAXI-2: State-Trait Anger Expression Inventory-2
